# Longitudinal detection of gait alterations associated with hypertension-induced cerebral microhemorrhages in mice: predictive role of stride length and stride time asymmetry and increased gait entropy

**DOI:** 10.1007/s11357-024-01210-3

**Published:** 2024-06-25

**Authors:** Zoltan Ungvari, Mihaly Muranyi, Rafal Gulej, Sharon Negri, Adam Nyul-Toth, Boglarka Csik, Roland Patai, Shannon Conley, Madison Milan, Jonathan Bagwell, Daniel O’Connor, Amber Tarantini, Andriy Yabluchanskiy, Peter Toth, Anna Csiszar, Anna Ungvari, Peter Mukli, Stefano Tarantini

**Affiliations:** 1 Vascular Cognitive Impairment, Neurodegeneration, and Healthy Brain Aging Program, Department of Neurosurgery, University of Oklahoma Health Sciences Center, Oklahoma City, OK USA; 2 Oklahoma Center for Geroscience and Healthy Brain Aging, University of Oklahoma Health Sciences Center, Oklahoma City, OK USA; 3 International Training Program in Geroscience, Doctoral School of Basic and Translational Medicine/Department of Public Health, Semmelweis University, Budapest, Hungary; 4 Stephenson Cancer Center, University of Oklahoma, Oklahoma City, OK USA; 5 Department of Public Health, Semmelweis University, Budapest, Hungary; 6 Department of Cell Biology, University of Oklahoma Health Sciences Center, Oklahoma City, OK USA; 7 Department of Neurosurgery, Medical School, University of Pecs, Pecs, Hungary; 8 International Training Program in Geroscience, Doctoral School of Basic and Translational Medicine/Department of Translational Medicine, Semmelweis University, Budapest, Hungary

**Keywords:** Gait coordination, Aging, Small vessel disease, Microbleed, Neurodegeneration, Motor performance, Balance, Intra-cerebral hemorrhage

## Abstract

Cerebral microhemorrhages (CMHs) are of paramount importance as they not only signify underlying vascular pathology but also have profound implications for cognitive function and neurological health, serving as a critical indicator for the early detection and management of vascular cognitive impairment (VCI). This study aimed to investigate the effects of hypertension-induced CMHs on gait dynamics in a mouse model, focusing on the utility of advanced gait metrics as sensitive indicators of subclinical neurological alterations associated with CMHs. To induce CMHs, we employed a hypertensive mouse model, using a combination of Angiotensin II and L-NAME to elevate blood pressure, further supplemented with phenylephrine to mimic transient blood pressure fluctuations. Gait dynamics were analyzed using the CatWalk system, with emphasis on symmetry indices for Stride Length (SL), Stride Time (ST), and paw print area, as well as measures of gait entropy and regularity. The study spanned a 30-day experimental period, capturing day-to-day variations in gait parameters to assess the impact of CMHs. Temporary surges in gait asymmetry, detected as deviations from median gait metrics, suggested the occurrence of subclinical neurological signs associated with approximately 50% of all histologically verified CMHs. Our findings also demonstrated that increases in gait entropy correlated with periods of increased gait asymmetry, providing insights into the complexity of gait dynamics in response to CMHs. Significant correlations were found between SL and ST symmetry indices and between these indices and the paw print area symmetry index post-hypertension induction, indicating the interdependence of spatial and temporal aspects of gait affected by CMHs. Collectively, advanced gait metrics revealed sensitive, dynamic alterations in gait regulation associated with CMHs, resembling the temporal characteristics of transient ischemic attacks (TIAs). This underscores their potential as non-invasive indicators of subclinical neurological impacts. This study supports the use of detailed gait analysis as a valuable tool for detecting subtle neurological changes, with implications for the early diagnosis and monitoring of cerebral small vessel disease (CSVD) in clinical settings.

## Introduction

Cerebral microhemorrhages (CMHs) represent a critical neuropathological condition characterized by small (< 5 mm), yet significant, areas of hemorrhage within the brain parenchyma [1–24]. CMHs are increasingly recognized for their contribution to the pathogenesis of vascular cognitive impairment and dementia (VCID), a spectrum of cognitive impairments linked to vascular pathologies.

Advanced age and hypertension are identified as the primary risk factors for CMHs [1, 20], with the prevalence of CMHs notably increasing with age—from approximately 6.5% in individuals aged 45 to 50 years to over 50% in older adults [20]. Recent data from rodent models further corroborate these clinical observations, highlighting a synergistic interplay between aging and hypertension in the genesis of CMHs [25]. As manifestations of cerebral small vessel disease (CSVD), CMHs share the pathological landscape with white matter hyperintensities (WMHs) and lacunes, painting a complex picture of cerebromicrovascular vulnerability.

The development of cerebral microhemorrhages is closely linked to microvascular fragility induced by aging, a process driven by increased vascular oxidative stress, microvascular senescence, and the secretion of activated matrix metalloproteinases [1, 25, 26]. The pathophysiological outcomes of hypertension-induced rupture of cerebral microvessels encompass a range of effects, such as the compression of neuronal tissue, regional ischemia, and subsequent ischemic neuronal damage [27]. These consequences collectively establish a foundation for the development of cognitive impairments.

Against this backdrop, gait analysis has emerged as a potent investigative tool in geroscience research, providing deep insights into the neurological and vascular health of older individuals [28–32]. The analysis of gait—the intricate pattern of limb movements during locomotion—reveals subtle yet significant changes in motor function that conventional diagnostic methods may overlook. This technique acts as a prism through which the nuanced relationship among central nervous system health, motor capabilities and cognitive health can be observed, making it an essential method for identifying and monitoring neurological conditions linked to vascular impairments.

In exploring the nexus between hypertension, CMHs, and gait alterations, our research has pioneered a mouse model that recapitulates CMHs induced by hypertension in aged mice [25, 26, 33–37]. This model has illuminated the exacerbating role of hypertension in the development of CMHs, a scenario further intensified by the microvascular aging process [25, 26]. A pivotal finding from our investigations is the strong association between an increased burden of CMHs and significant alterations in mouse gait, suggesting the utility of gait analysis as a sensitive early indicator of VCID, especially prominent in the context of aging [25, 26].

Building on the existing body of research that underscores gait alterations as markers of age-related cognitive decline in humans [28–32, 38–43], our study embarked on a foundational journey to longitudinally trace gait alterations linked to the hypertension-driven genesis of CMHs. We hypothesized that temporal variations in gait metrics, particularly those that encapsulate higher-level coordination—such as stride length and stride time symmetry, gait entropy, and the regularity of paw placements—may serve as reliable predictive indicators of CMHs. This concept is supported by our previous findings, which establish a compelling correlation between gait alterations and the occurrence of CMHs under hypertensive conditions in mice [25, 26, 36]. To validate our hypothesis, hypertension was induced in 8-month-old mice through the administration of angiotensin II and the NO synthase inhibitor L-NAME, complemented by phenylephrine injections to simulate transient blood pressure fluctuations. Throughout the 30-day experimental period, gait function was analyzed daily in freely walking mice to detect day-to-day fluctuations in stride time, stride length, and paw print areas using the semi-automated, highly sensitive, Catwalk XT system. We adopted and developed methods to analyze stride time, stride length symmetry, and gait entropy, which are considered sensitive indices of CSVD-related subclinical gait abnormalities in humans [28]. At the conclusion of the experiment, histological analysis was conducted to confirm the presence of CMHs in the brains of the mice.

## Methods

### Experimental animals

All experimental procedures involving animals, as well as animal husbandry practices, were conducted with the approval of the Institutional Animal Care and Use Committee (IACUC) at the University of Oklahoma Health Sciences Center (OUHSC), OK, USA. In this study, we utilized a cohort of male C57BL/6 mice (8-month-old, *n* = 20) procured from Charles River Laboratories, Wilmington, MA. Mice were housed in the Rodent Barrier Facility at OUHSC under specific pathogen-free barrier conditions and a controlled photoperiod (12 h light; 12 h dark). Mice were provided with unrestricted access to standard rodent chow (sourced from Purina Mills, Richmond, IN, USA) and water.

### Induction of spontaneous CMHs

To investigate the longitudinal effects of hypertension-induced CMHs on gait coordination, we employed a well‐characterized mouse model [25, 26]. Briefly, in 8‐month‐old male C57BL/6 mice (*n* = 20), hypertension was induced by administering a combination treatment of the nitric oxide inhibitor ω-nitro-L-arginine-methyl ether (L-NAME, 100 mg/kg/day) in drinking water, along with subcutaneous administration of angiotensin II (Ang-II; s.c. via osmotic mini-pumps [Alzet Model 2006, 0.15 µl/h, 42 days; Durect Co, Cupertino, CA]), supplemented with phenylephrine (PE, 65 mg/kg/day, administered subcutaneously daily) to mimic transient blood pressure fluctuations experienced in daily human life. The osmotic pumps, filled with angiotensin II (Sigma Chemical Co., St. Louis, MO, USA), delivered 1 μg/min/kg of angiotensin II subcutaneously throughout the entire experimental period of 30 days. The chosen dosages of L-NAME and Ang-II were based on the outcomes of prior studies utilizing the same protocol [25, 44]. The implantation of the pumps was performed under isoflurane anesthesia. A small incision was made in the interscapular area of the mice to place the pumps in the subcutaneous space, which was then closed with surgical sutures using aseptic techniques. Post-surgery, buprenorphine analgesia (1 mg/kg of body weight, subcutaneously) was administered to manage pain. The incision sites healed promptly without necessitating additional treatment. Throughout the treatment period, the blood pressure of the animals was monitored using a tail-cuff blood pressure system (CODA Non-Invasive Blood Pressure System, Kent Scientific Co., Torrington, CT) [25, 45, 46] to confirm successful induction of hypertension.

### Standardized neurological examination of mice

We carried out a standardized neurological examination on the mice to assess for clinical indicators of hemorrhages, a crucial aspect of our study on the effects of hypertension-induced cerebral microhemorrhages [25, 26]. This comprehensive examination evaluated several key areas: the animals’ spontaneous activity, symmetry in movement of all four limbs, ability to outstretch forelimbs, climbing proficiency, body proprioception, response to vibrissae (whisker) touch, and overall gait coordination. To systematically gauge the neurological health of each mouse, a daily score was assigned based on the cumulative results from all the individual tests conducted. This structured approach allowed for a nuanced assessment of the mice’s neurological function over time. In instances where a consistent decline in the neurological score was observed, indicating a potential exacerbation of brain hemorrhages or other serious neurological impairments, the mice were humanely euthanized to prevent further suffering. Following euthanasia, the brains were collected after transcardial perfusion.

### Analysis of gait function

To assess the day-to-day variations in gait function and to analyze the spatial and temporal dimensions of interlimb coordination, we utilized the CatWalk System (Noldus Information Technology Inc., Leesburg, VA). This approach, following the established methodology [25, 36, 37, 47–51], allows for a detailed quantification and visualization of gait dynamics, offering insights into how CMHs affect locomotor behavior over time. Briefly, animals from each group were acclimatized and trained to voluntarily walk across the illuminated walkway in a dark and quiet room dedicated for behavioral experimentation. Gait function in mice was acquired for over 20 consecutive runs, during which the mice maintained a relatively constant speed across the walkway, producing over 200 steps per animal. Subsequently, computer-aided analysis of the gait data and manual paw identification and labeling of each footprint was carried out blindly, and spatial and temporal gait parameters were calculated [36]. Gait in each mouse was analyzed on the day before hypertension induction and each day thereafter. This allowed us to assess changes in various gait parameters associated with CMHs.

The variability within our dataset was meticulously evaluated through quartile dispersion, a robust measure of spread that provides insight into the data's distribution without being overly influenced by extreme values. Following a widely accepted practice for identifying outliers, we classified any data points that lay more than 1.5 interquartile ranges (IQRs) from the sample’s median as extreme values. This approach ensures a consistent method for delineating the central tendency and variability of our data, aiding in the accurate interpretation of gait characteristics. The mean gait characteristics analyzed in this study encompassed stride length, stride time, and paw print areas. Specifically, stride length refers to the distance in centimeters (cm) measured between the successive placements of the same paw, providing a direct measure of the spatial aspect of gait. Stride time, measured in seconds (s), denotes the duration elapsed between consecutive contacts of the same paw with the ground, offering insights into the temporal dimension of locomotion.

The regularity index (%) is a quantitative metric designed to assess inter-paw coordination during locomotion. It calculates the proportion of normal step sequence patterns observed in relation to the total number of paw placements. This measure offers a precise evaluation of the gait’s rhythmicity and coordination, providing insights into the functional integrity of the locomotor system. By highlighting deviations from normal gait patterns, the regularity index serves as an invaluable tool in the detection and characterization of alterations in gait dynamics, potentially resulting from neuronal damage associated with CMHs. In healthy, fully coordinated mice, the regularity index value approaches 100%, indicating a high level of inter-paw coordination and the prevalence of normal step sequence patterns. This high percentage reflects an optimal gait pattern where the timing and placement of paw contacts are consistent and rhythmic, signifying the intact neurological function.

1 $$\text{Regularity Index}= \frac{\#\text{ of normal step sequence patterns }\times 4 }{\text{total }\#\text{ of paw placements}}\times (100\%)$$

### Analysis of gait symmetry

Analyzing gait symmetry offers a nuanced and sensitive method for evaluating gait coordination. Given the often asymmetric locations of cerebral microhemorrhages, assessing gait symmetry can be particularly revealing, potentially uncovering subtle, sub-clinical changes in gait coordination that may not be immediately apparent [36]. This study delved into the impact of CMHs on gait symmetry by calculating the symmetry index (SI), a measure that has gained recognition for its sensitivity and widespread application in assessing gait symmetry based on spatial–temporal characteristics of movement.

The symmetry index provides a quantitative framework to evaluate the equivalence of gait parameters between the left and right limbs, thereby offering insights into the balance and coordination of gait [50, 52]. The formula used to calculate the Stride Length (SL) symmetry index serves as an example of this approach.

2 $$\text{Symmetry Index}=\frac{\left|{SL}_{Left}-{SL}_{Right}\right|}{0.5 \times ({SL}_{Left}+{SL}_{Right})}\times (100\%)$$

This calculation was mirrored for other critical gait parameters, including Stride Time and Paw Print Area, allowing for a comprehensive analysis of gait symmetry across multiple dimensions. Such detailed quantification of symmetry indices is instrumental in identifying the subtle effects of CMHs on gait dynamics, shedding light on the broader implications of cerebral microvascular alterations on locomotor behavior. The symmetry index serves as a method for evaluating the percentage differences between limbs in terms of kinematic and kinetic parameters during locomotion, offering a nuanced view of gait symmetry. An SI value of 0 denotes perfect symmetry, indicating that there is no discernible difference in the gait parameters between the left and right limbs. Conversely, an SI value of 100% or greater reflects a complete lack of symmetry, signifying pronounced disparities in the gait characteristic being analyzed.

### Analysis of gait entropy

The concept of entropy, which offers a way to quantify the disorder or unpredictability within a system, can be applied to a variety of physiological processes in a model-free manner [53], including the dynamics of gait. This approach provides a framework for assessing the complexity and regularity of gait patterns, which can be particularly useful in identifying deviations from typical gait due to neurological impairments, including neurological damage associated with CSVD in humans [28].

Among the techniques developed to estimate entropy from gait sequence data [54, 55], sample entropy (SE) and approximate entropy (AE) are noteworthy. Both methods are attuned to the length of the signal [56, 57], with their sensitivity to this parameter influencing their utility in analyzing gait dynamics. SE and AE have been utilized to measure the amount of regularity and the unpredictability of fluctuations over time within gait cycles, offering insights into the gait’s stability and coordination. Expanding upon the principles of AE and SE, Bandt and Pompe [58] introduced permutation entropy (PE) as a method that is particularly suited for analyzing short datasets [59]. PE distinguishes itself by simplifying the computational model, requiring only two input parameters: the embedding dimension (*m*) and the time lag (*L*). Therefore, the implementation of PE for gait analysis is straightforward [28], providing a robust measure of gait dynamics’ entropy even when data availability is limited. The adoption of PE in gait analysis underscores the ongoing evolution of methodologies for understanding the intricacies of locomotor function and its alterations across different conditions.

The calculation of PE initiates with embedding a discretely sampled time series $x\left(t\right)={x}_{1},{x}_{2},\dots ,{x}_{N}$ of length *N* –– in an $m$-dimensional state space. This process is achieved through the construction of a sequence of state space vectors $X(t)$ utilizing a temporal embedding scheme [60]. Specifically, when using an embedding time lag *L*, and embedding dimension $m$, $X(t)$ is defined as

3 $$X\left(t\right)=\left[{x}_{t}, {x}_{t-L}, \dots , {x}_{t-\left(m-1\right)L}\right]$$

Then values in each embedding vector $X(t)$ are converted to ranks, converting $X(t)$ (which consists of $m$ data points) into one of the $m!$ possible permutations of rank $m$. The relative frequency of each permutation/order sequence is calculated as

4 $$p\left(\pi \right)=\frac{\# \;of \;type \;\pi \;permutation}{T-\left(m-1\right)L+1}$$

yielding an estimate on the probability distribution of each permutation type in the process [61, 62]. Finally, permutation entropy of order $m$, $H(m)$ is defined [61] as

5 $$H\left(m\right)=-\sum_{\pi \in\Pi }p\left(\pi \right)\text{log}p(\pi )$$

where $\Pi$ denotes the set of $m!$ possible permutations of rank$m$. Based on the concept of information theoretical entropy and Eq. (4), $H(m)$ has a theoretical maximum of $\text{log}m!$; which is the case when all permutations occur with equal probability that corresponds to maximal entropy [61]. Therefore, PE can be ‘normalized’ to be in the range$[0, 1]$, where 0 and 1 indicate complete predictability (minimal complexity) and maximal unpredictability (maximal complexity), respectively. In most studies, the input parameters $m$ and $L$ of PE are set according to empirical or practical considerations rather than being defined by the characteristics of the data itself [63, 64]. In case of human gait, the typical value of *m* is 3 and *L* is 1 [59, 65].

### Histological analysis of CMHs

Mice were anesthetized and underwent transcardial perfusion with ice-cold heparinized phosphate-buffered saline (PBS) for 10 min, a process designed to clear the blood while preserving tissue integrity, followed by humane decapitation as reported [25, 36]. Subsequently, their brains were carefully extracted from the skull and initially fixed in 4% formalin at room temperature for 24 h. The following day, to ensure thorough fixation, the brains were transferred to fresh 4% formalin, chilled to 4 °C, for an additional 48 h, and then submerged in 70% ethanol at 4 °C for 2 days to dehydrate the tissue, followed by embedding in paraffin. The brains were serially sectioned at 8 μm thickness yielding approximately 800 sections per brain. The sections were stained with hematoxylin to reveal the brain structure and diaminobenzidine (DAB) to highlight the presence of hemorrhages. DAB reacts with peroxidases in red blood cells, turning dark brown, thus enabling the precise identification of extravasated blood cells in the parenchyma of the brain. A blinded reviewer meticulously examined all stained sections, capturing images in areas where DAB staining indicated hemorrhages. Digital images were analyzed with ImageJ 1.52p (NIH, USA) software as described [26].

### Statistical analysis

Two-tailed *t*-test was used for comparison of two groups. Two-way Analysis of Variance followed by Fisher LSD method or Kruskal–Wallis One Way Analysis of Variance on Ranks was used for comparison of multiple groups as needed based on the sample distribution. The incidence curves were compared with Mantel-Cox log-rank test. A *P* value < 0.05 was considered statistically significant. Data are expressed as mean ± SEM.

## Results

### Hypertension-induced CMH mouse model

**Fig. 1:**
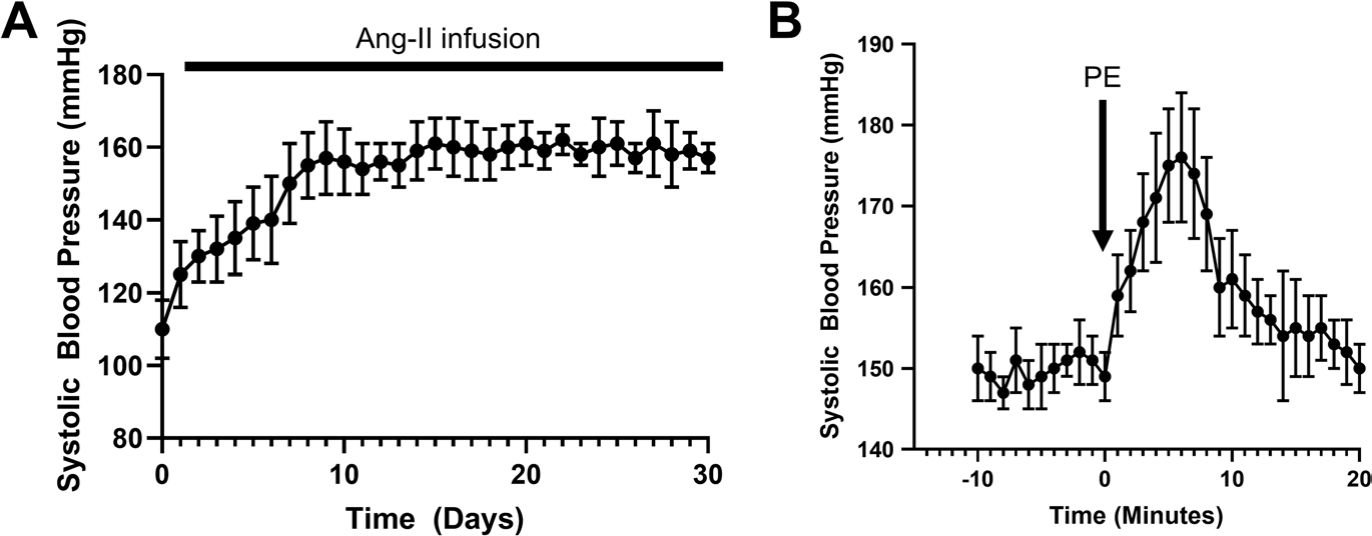
Monitoring of systolic blood pressure in a model of hypertension-induced genesis of cerebral microhemorrhages. **A** Treatment with angiotensin II plus L‐NAME elicited an increase in systolic blood pressure during the 30-day experimental timeline. **B** Acute systolic blood pressure spikes were obtained by subcutaneous injection of phenylephrine (PE). Data are mean ± SEM, *n* = 20

The treatment regimen combining Angiotensin II (Ang II) with L-NAME led to notable elevations in blood pressure across the 30-day experimental period, as illustrated in Fig. 1A. This sustained increase in blood pressure underscores the efficacy of the combined Ang II and L-NAME treatment in inducing a hypertensive state in the experimental animals. Additionally, Fig. 1B highlights the occurrence of acute systolic blood pressure spikes, which were elicited through the subcutaneous administration of phenylephrine. These acute fluctuations in blood pressure mimic the transient spikes observed during daily activities in human hypertension.

**Fig. 2:**
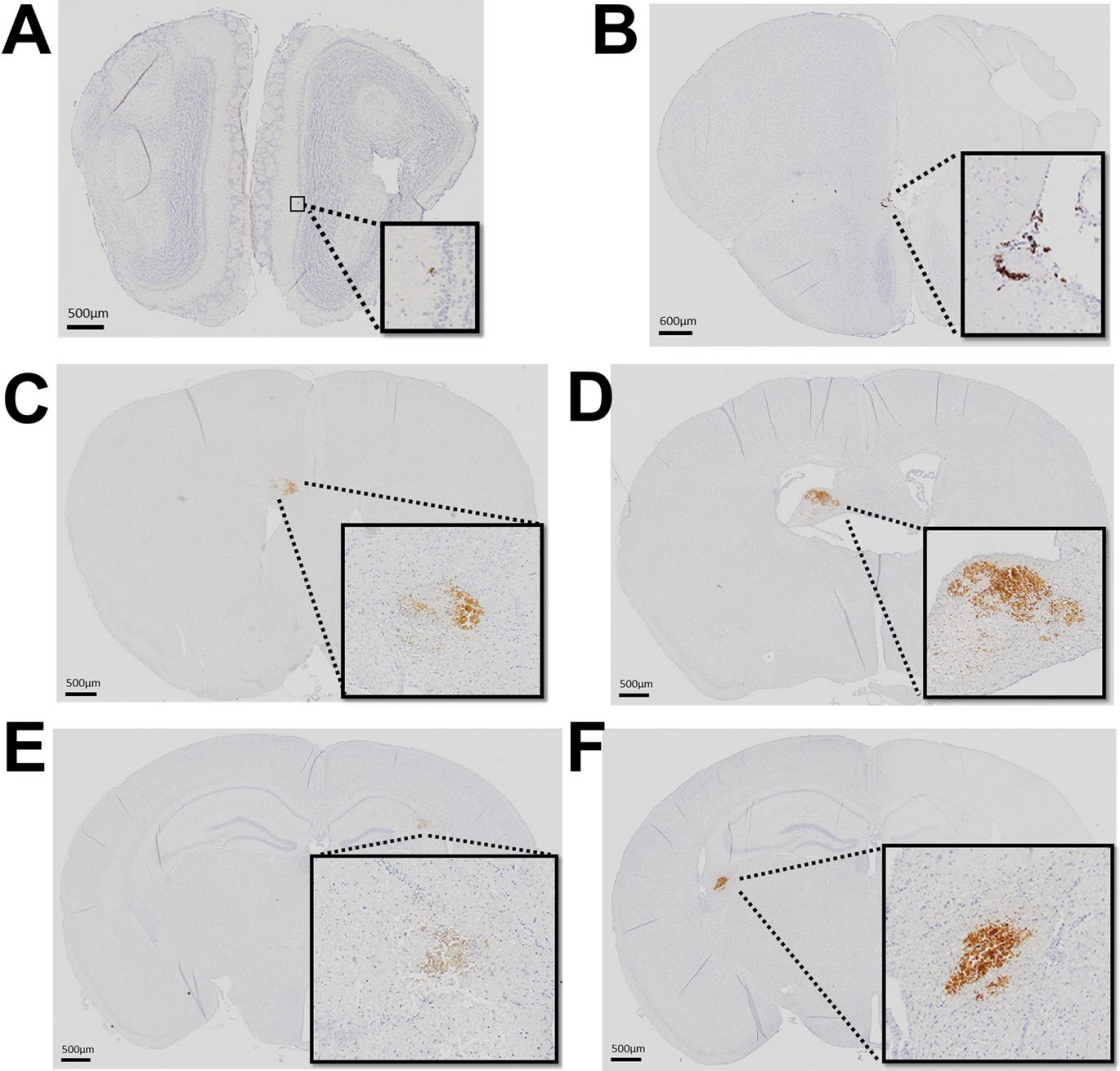
Distribution of cerebral microhemorrhages in the brain of a hypertensive mouse. **A**–**F** Representative diaminobenzidine-stained images showcasing cerebral microhemorrhages within various regions of the cortex in a hypertensive mouse. Brightfield microscopy was utilized to capture the CMHs, employing both 10 × and 4 × objectives to detail their extensive distribution across the brain. Panels **A** and **B** depict small bleeds located in the olfactory bulb, Panels **C** and **D** show CMHs within the white matter, in corpus callosum (**C**) and fimbria of the hippocampus (**D**). Panel **E** features a CMH in the right hippocampus, a region critical for memory and spatial navigation, indicating the potential impact on cognitive functions. Panel **F** presents a CMH within the white matter of the left hemisphere, underscoring the vulnerability of both grey and white matter to hypertension-induced hemorrhagic damage. Images from panels **A-E **highlight the widespread nature of hemorrhagic events across different functional areas of the brain. This brain is from the same mouse whose altered gait patterns following hypertension induction are depicted in Fig. 4, linking structural brain changes with functional locomotor deficits

Histological examination conducted at the conclusion of the experimental timeline revealed that induction of hypertenstion in mice led to the widespread formation of CMHs across various regions of the brain, as depicted in Fig. 2. In contrast, mice maintained under normotensive conditions did not exhibit any histologically identifiable CMHs, underscoring the direct correlation between hypertension and the development of CMHs (data not shown).

### Temporal dynamics of gait alterations following hypertension induction in mice

**Fig. 3:**
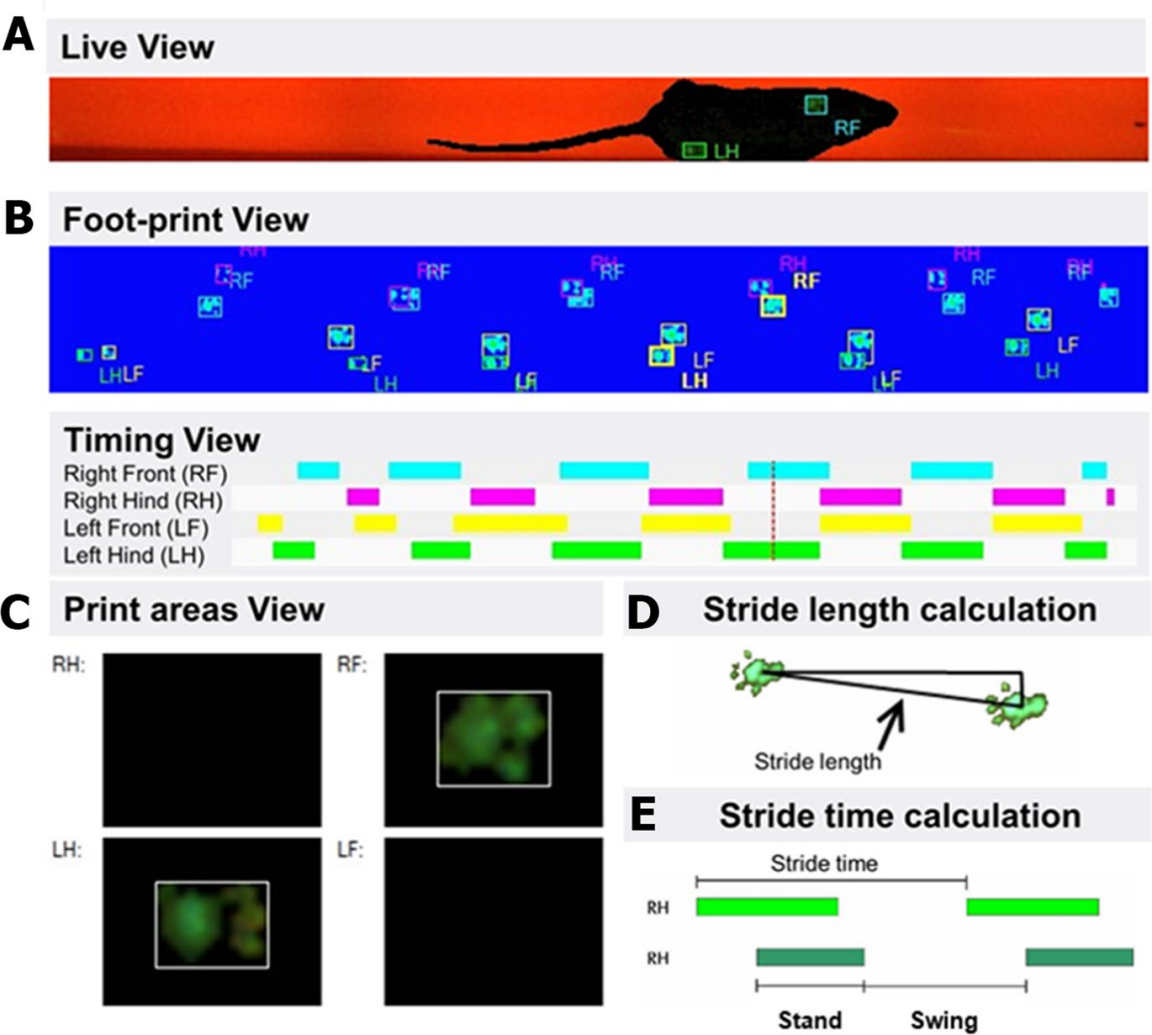
Gait analysis in mice utilizing the CatWalk system. **A** Footprint view illustrating the placement of paws on the glass walkway, crucial for assessing spatial gait patterns. **B** Timing view depicting the duration of each paw’s contact with the glass, instrumental for analyzing footfall patterns and the calculation of the regularity index. **C** Print areas, highlighting the spatial dimensions of paw contacts. **D** Calculation of stride length, detailing the measurement of distance between successive placements of the same paw. **E** Calculation of stride time, indicating the time elapsed between consecutive paw contacts

Figure 3 offers a comprehensive overview of the gait analysis conducted on mice using the CatWalk system, a sophisticated tool designed to capture and analyze the intricacies of animal locomotion. This system allows for a detailed examination of gait dynamics through various lenses. Figure 3A presents the placement of paws on the glass walkway, a fundamental aspect for evaluating spatial gait patterns. By visualizing the exact footprints left by the mice, we can assess stride length, base of support, and other spatial parameters critical to understanding locomotion. Figure 3B focuses on the duration of paw contact with the glass surface. It is essential for analyzing footfall patterns and plays a crucial role in the calculation of the regularity index, offering insights into the temporal aspects of gait that reflect the coordination between movements of different limbs. Figure 3C highlights the spatial dimensions of paw contacts. This aspect of the analysis provides information on the area covered by each paw during contact with the walkway. Figure 3D illustrates calculation of stride length. This component details the measurement of distance between successive placements of the same paw, a critical parameter for assessing the stride characteristics and spatial aspects of gait. Figure 3E illustrates calculation of stride time. Indicating the time elapsed between consecutive paw contacts, this measurement offers insights into the pace and rhythm of the animal’s gait, contributing to our understanding of the temporal dynamics of locomotion. An essential consideration in such gait analyses is the walking speed of the subjects, as variations in velocity can significantly influence gait patterns. The CatWalk system accommodates this by allowing mice to traverse the walkway at their preferential speed, thereby ensuring that the observed gait differences are attributable to neuronal coordination rather than differences in walking speed. The data collected from these experiments clearly show that body speeds were uniformly maintained across all animals and experimental days, reinforcing the conclusion that any observed variations in gait are due to changes in neuronal coordination and not the speed at which the animals moved.

**Fig. 4:**
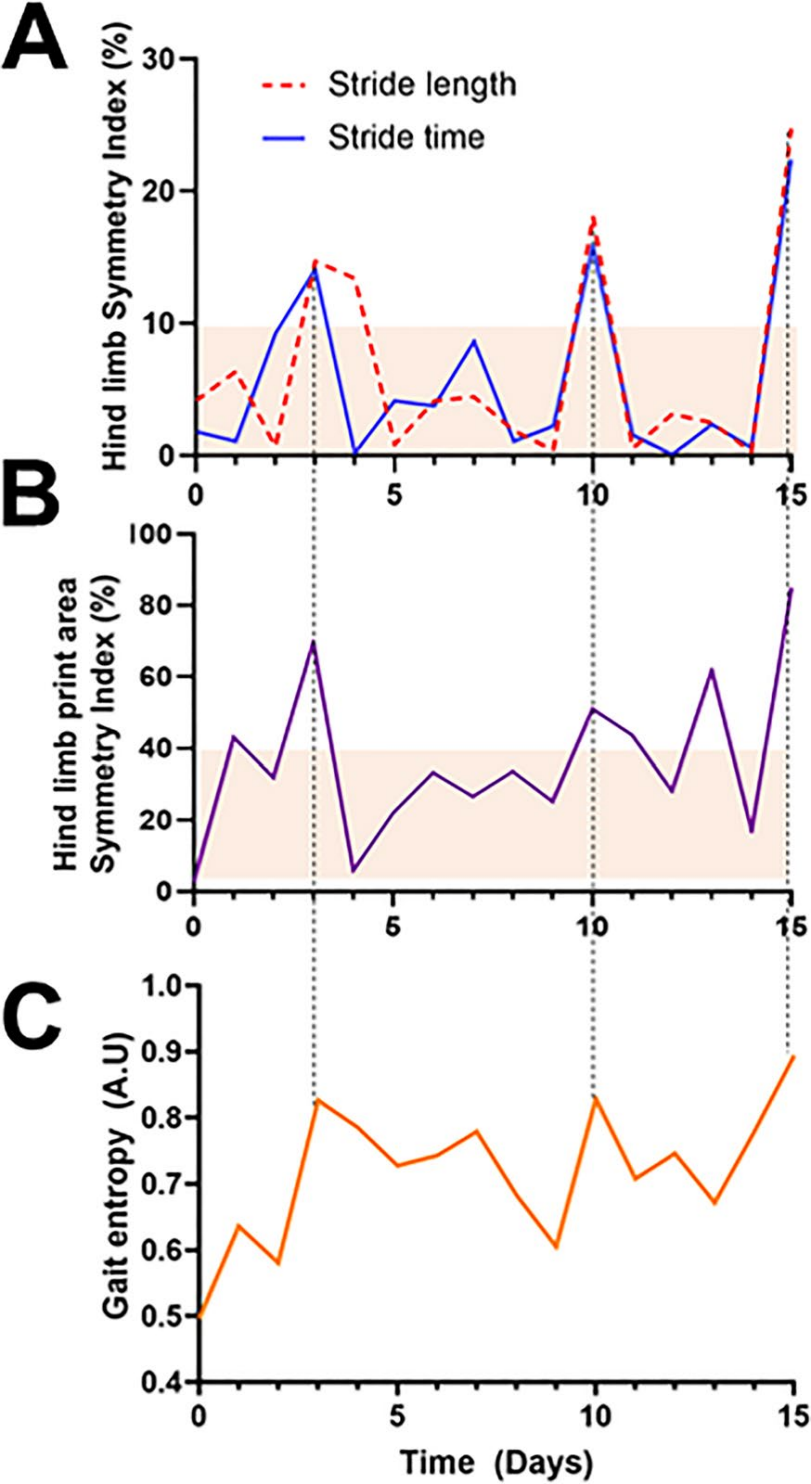
Temporal dynamics of gait alterations following hypertension induction in mice. **A** Day-to-day variations in hind limb stride length and stride time symmetry indices after hypertension induction, highlighting temporary surges in interlimb gait asymmetry lasting 1–2 days. This analysis corresponds to the same mouse whose brain, exhibiting cerebral microhemorrhages, is presented in Fig. 2. **B** Day-to-day fluctuations in the hind limb paw print area symmetry index following hypertension induction, illustrating periods of print area asymmetry that coincide with the surges observed in SL and ST asymmetry. **C** Day-to-day changes in gait entropy following hypertension induction, with evident increases corresponding to periods of increased gait asymmetry, providing insights into the temporal dynamics of CMH-induced subclinical neurological symptoms

Our examination of the longitudinal effects of hypertension induction on gait dynamics in mice, as depicted in Fig. 4, elucidates the intricate temporal patterns of gait alterations associated with CMHs. In our analysis, “surges” in gait parameters were defined with a specific statistical approach to ensure objective identification of significant alterations over time. For each time series—comprising data on stride length and stride time symmetry indices, the paw print area symmetry index, and gait entropy—a median value was first calculated to establish a central tendency around which normal fluctuations were expected to occur. A “surge” was defined as any value exceeding the threshold that contains 95% of all values, indicating a significant deviation from typical patterns. This methodological criterion was chosen to distinguish between normal day-to-day variability and significant deviations indicative of a surge in gait asymmetry or entropy. By setting this threshold, we aimed to accurately identify and quantify episodes of pronounced change in gait dynamics that could be directly associated with the physiological and neurological impact of hypertension-induced CMHs. This rigorous definition of a “surge” allowed us to systematically identify and analyze the instances of acute and temporary gait asymmetry, providing insights into the temporal dynamics of CMH-induced subclinical neurological symptoms and their manifestation in locomotor function.

Figure 4A showcases representative day-to-day variations in hind limb stride length and stride time symmetry indices following hypertension induction. Notably, we observed temporary surges in interlimb gait asymmetry, lasting between 1 and 2 days. These fluctuations in SL and ST indices highlight the immediate impact of CMHs on gait dynamics. Figure 4B reveals day-to-day fluctuations in the hind limb paw print area symmetry index post-hypertension induction. It is evident that the observed periods of print area asymmetry align closely with the surges noted in SL and ST asymmetry. This congruence underscores the broader impact of CMH-induced neurological changes on various aspects of gait, from stride dimensions to the spatial characteristics of paw placement.

Finally, Fig. 4C focuses on the day-to-day changes in gait entropy following hypertension induction. The evident increases in gait entropy correspond to periods of enhanced gait asymmetry, shedding light on the heightened variability and complexity in locomotion induced by CMHs. This suggests that hypertension not only alters the regularity and symmetry of gait but also introduces significant variability into locomotor patterns, likely reflecting the underlying subclinical neurological symptoms induced by CMHs.

Intriguingly, the analysis we presented corresponds to the same mouse featured in Fig. 2, which exhibited six evident cerebral microhemorrhages upon histological examination. Notably, in this particular animal, we identified three significant surges in gait indices within the time series data collected. This pattern of surges in response to CMHs was not isolated to this mouse alone but was similarly observed across other mice in the experimental group, suggesting a consistent phenomenon. The detection of three surges in the context of six CMHs leads us to interpret that our analysis method is sensitive to capturing subclinical neurological signs associated with approximately 50% of all CMH occurrences. This rate of detection underscores the potential of gait analysis as a tool for identifying neurological alterations that may not present overt clinical symptoms but nonetheless reflect underlying brain injuries. Furthermore, our findings hint at the complexity of the relationship between the physical characteristics of CMHs and their impact on gait. We hypothesize that the specific manifestations of gait alterations are influenced by both the location and the size of the CMHs. This suggests that the precise impact of CMHs on locomotion may vary depending on how these microhemorrhages interact with neural circuits involved in motor function and coordination. Therefore, the variations in gait dynamics we observed could reflect the differential impact of CMHs on these circuits, pointing to the nuanced ways in which microvascular brain damage can influence overall motor behavior.

**Fig. 5:**
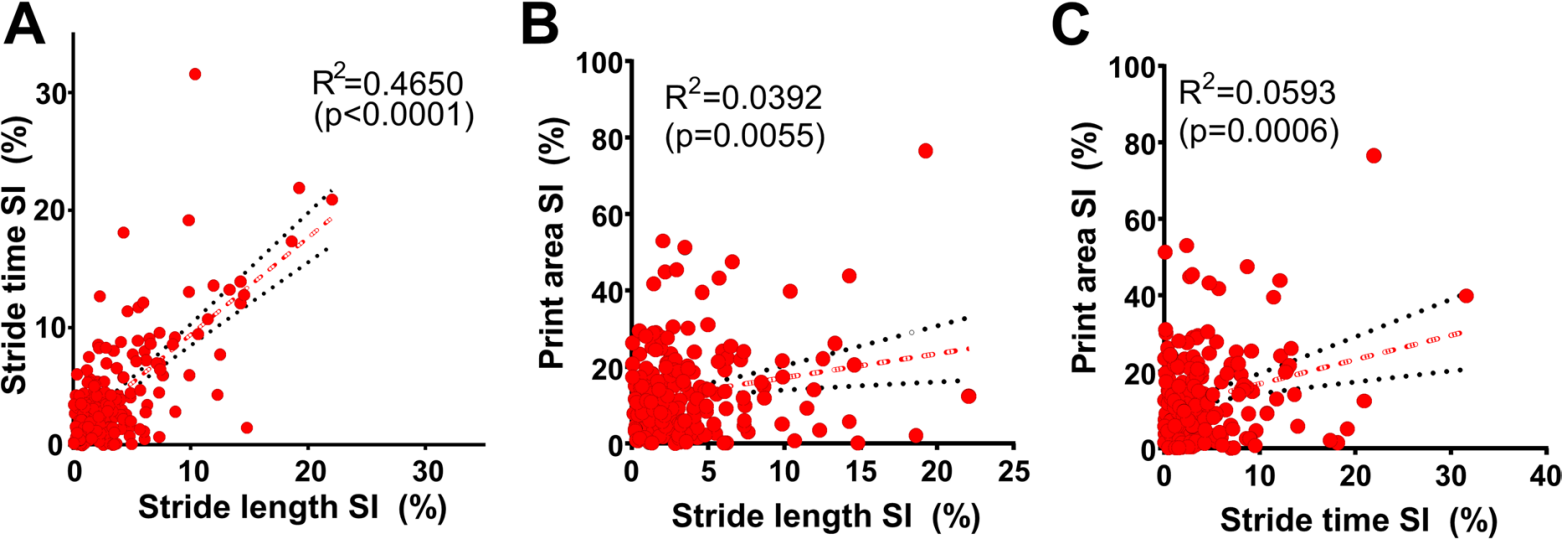
Correlations between gait symmetry indices in hypertensive mice. Shown are the interrelationships among various gait symmetry indices in mice following hypertension induction, capturing each time point throughout the 30-day experimental period for each mouse (*n* = 20). **A**) Correlation between stride time symmetry index and stride length symmetry index post-hypertension, highlighting the strongest correlation observed among the indices. This strong correlation underscores the interdependence of temporal and spatial aspects of gait symmetry affected by CMHs. **B**) Correlation between the paw print area symmetry index and SL symmetry index in mice post-hypertension, illustrating how changes in the spatial domain of gait (SL and print area) are interrelated following hypertension induction. **C**) Correlation between the paw print area symmetry index and ST symmetry index post-hypertension induction, demonstrating the relationship between the spatial domain of print area and the temporal aspect of gait (ST), further emphasizing the comprehensive impact of CMHs on gait dynamics. Each panel provides insight into how CMHs affect the interplay of different gait parameters, with each point representing a day in the life of the hypertensive mice, reflecting the dynamic nature of gait alterations over the course of the experimental period

Figure 5 delves into the intricate correlations between various gait symmetry indices in mice subjected to hypertension induction, presenting a detailed view of the interplay among these indices across the entire 30-day experimental timeline for each of the 20 mice studied. This analysis revealed a pronounced correlation between the stride time symmetry index and the stride length symmetry index following hypertension induction (Fig. 5A), marking the strongest correlation observed among the examined indices. The robustness of this correlation highlights a significant interdependence between the temporal and spatial components of gait symmetry, which are collectively influenced by the presence of CMHs. This finding suggests that alterations in stride time are closely linked to changes in stride length, reflecting the comprehensive impact of CMHs on the coordination of gait. Further analysis indicated a weaker correlation between the paw print area symmetry index and the SL symmetry index (Fig. 5B). This correlation underscores how spatial aspects of gait—both in terms of stride length and the area covered by each paw—are interconnected and altered in response to CMHs. Lastly, the study demonstrated a weak, yet significant relationship between the paw print area symmetry index and the ST symmetry index following hypertension induction (Fig. 5C). This correlation illuminates the link between the spatial domain of the print area and the temporal dynamics of gait, further reinforcing the notion that CMHs exert a widespread effect on both the timing and spatial characteristics of gait. Our analysis sheds light on the evolving landscape of gait dynamics under the influence of neurological changes, offering a window into the complex interrelations among different gait parameters in the face of CMH-induced alterations.

### Cumulative incidence of neurological signs

**Fig. 6:**
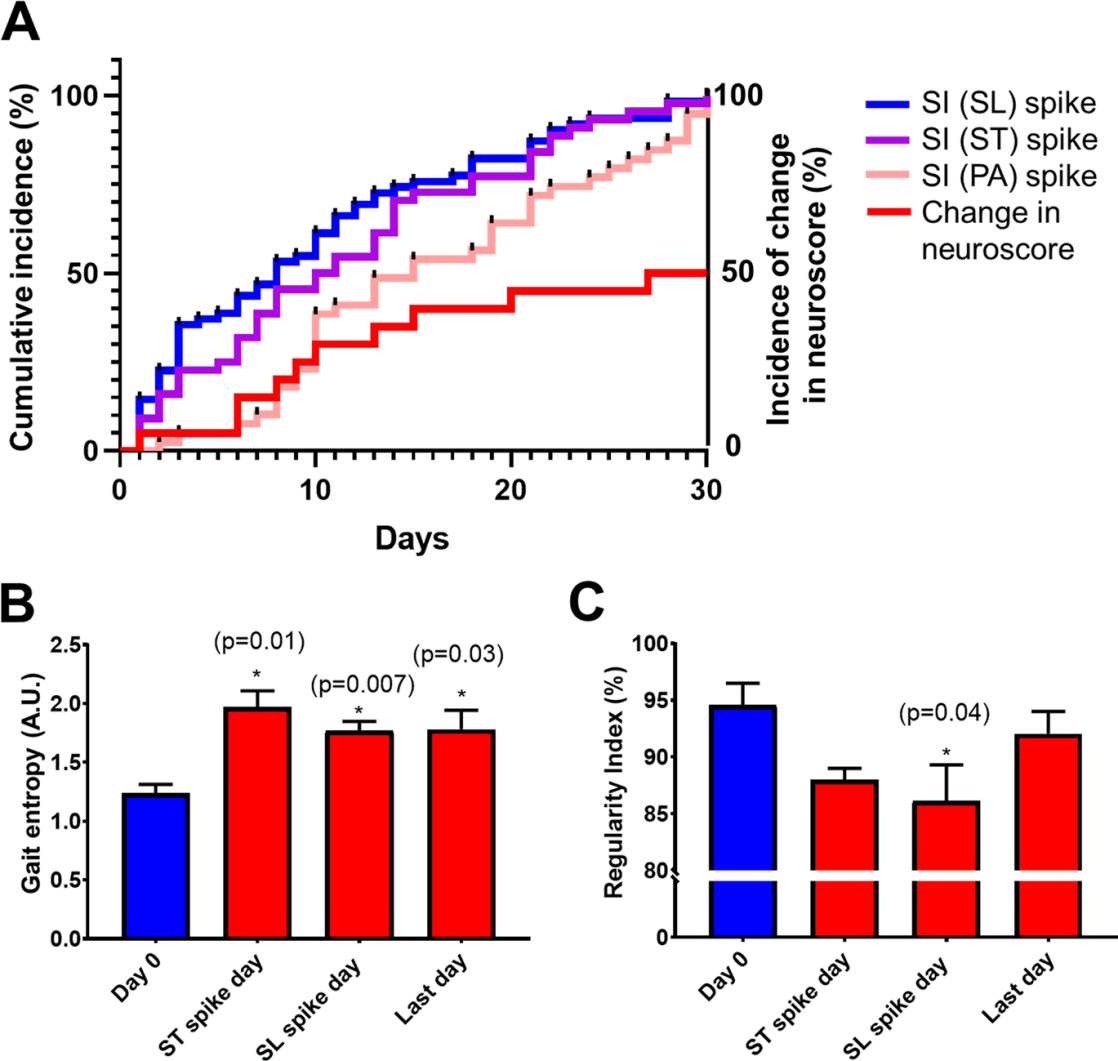
Neurological signs and gait dynamics following hypertension induction in mice. This figure provides a comprehensive overview of the progression of neurological signs and alterations in gait dynamics in response to hypertension-induced cerebral microhemorrhages in a cohort of 20 mice. **A**) The panel illustrates cumulative incidence curves for neurological signs of hypertension-induced CMHs, focusing on temporal surges (“spikes”) in gait asymmetry measured by the stride length (SL) symmetry index (SI), stride time (ST) SI, and print area (PA) SI, alongside the time-to-change curve for alterations in neuroscore. It is observed that detectable surges in SI for SL and ST precede those observed in the SI (PA), indicating that SL and ST indices are the most sensitive indicators for the detection of CMHs. Detection of changes in neuroscore demonstrated the least sensitivity, marking it as a less immediate indicator of CMH impact on neurological function. **B**) Variations in gait entropy at baseline (day 0, before hypertension induction), on days corresponding to identified surges in SI (ST) and SI (SL), and on day 30, the final day of the experimental timeline. **C**) Regularity Index measurements are shown for the same time points as in panel **B**, offering insights into inter-paw coordination at baseline, during surges in SI (ST) and SI (SL), and at the study's conclusion. Data are presented as mean ± SEM for the 20 mice studied

The analysis of cumulative incidence curves for neurological signs post-hypertension induction delineated a clear temporal pattern in the emergence of gait asymmetries (Fig. 6A). Specifically, surges in the Symmetry Index (SI) for Stride Length (SL) and Stride Time (ST) were observed to precede those in the Print Area (PA) SI. This pattern indicates that SL and ST indices are markedly sensitive to the early detection of CMHs, highlighting their utility in monitoring the initial impact of CMH-induced neurological changes. Conversely, surges in SI (PA) were detected only later, suggesting less sensitivity. Notably, changes in the neurological scores emerged as the least sensitive method for detecting the onset of CMH-related neurological alterations, pointing to the superior sensitivity of gait asymmetry indices in capturing the early stages of CMH impact.

Parallel analyses of gait entropy (Fig. 6B) and the Regularity Index (Fig. 6C) provided further insight into the dynamic changes in locomotor behavior induced by CMHs following hypertension induction. Gait entropy measurements taken at baseline (day 0, before hypertension induction), on days corresponding to identified surges in ST and SL, and on the final day of the experimental period (day 30) illustrated significant fluctuations. These changes underscore the variability in gait dynamics, reflecting the disorder introduced into locomotion by CMHs. Similarly, Regularity Index values across the same time points elucidated variations in inter-paw coordination, with noticeable deviations from baseline coordination patterns on days marking ST and SL surges, and at the study’s conclusion. Together, these findings underscore the profound impact of hypertension-induced CMHs on gait dynamics and neurological function in mice. The early and pronounced alterations in SL and ST symmetry indices, coupled with subsequent changes in print area symmetry and overall gait entropy and regularity, highlight the potential of these gait parameters as sensitive and early indicators of CMH-induced neurological alterations.

## Discussion

Our study systematically explored the impact of hypertension-induced CMHs on gait dynamics in mice, leveraging detailed analyses of gait symmetry indices and entropy measures to uncover the nuanced effects of CMHs on locomotion. The findings illuminate the intricate relationship between CMHs and altered gait patterns, underscoring the potential of gait analysis as a sensitive tool for detecting subclinical neurological signs associated with CMHs.

The design of the present study primarily addressed acute changes in gait following the induction of CMHs. This focus builds upon and extends our previous research, which detailed the long-term neurological consequences of CMHs within the same animal model [25, 26, 37]. Our observations of temporal surges in gait asymmetry and disorderliness in hypertensive mice have led to intriguing insights into the neurobiological underpinnings of CMHs and their systemic effects. This work builds upon our previous study investigating the temporal dynamics of neuronal tissue compression and microvascular constriction associated with laser-induced CMHs in the mouse brain [26]. We discovered that even a small CMH can exert a disproportionately large effect on surrounding brain tissue [26]. The dislocation of tissue and the strong, spreading microvascular constriction likely induce temporary ischemia and alterations in the humoral milieu, disrupting neuronal communication over a significantly larger area than the hemorrhage itself [26]. This phenomenon is akin to the concept of the stroke penumbra observed in larger ischemic strokes [66–68], where a core area of irreversible injury is surrounded by a zone of potentially salvageable tissue that is functionally impaired but not yet dead. In the context of CMHs, these “ministrokes” likely create a transient penumbra-like effect, where the immediate vicinity of the hemorrhage experiences a temporary disruption in normal neuronal activity and blood flow. However, as our findings suggest, the effects of tissue compression, vascular constriction, edema, and ischemia are likely reversible, which accounts for the resolution of observed neurological symptoms within a day or two. This transient nature of symptoms mirrors the clinical presentation of transient ischemic attacks (TIAs), often considered “warning strokes,” where neurological function is temporarily lost due to brief disruptions in blood supply but typically recovers quickly, usually within 24 h. The parallel between the temporal nature of symptoms attributed to TIAs and some of the neurological signs associated with experimentally induced CMHs raises an important consideration: that some transient neurological symptoms attributed to TIAs may, in fact, result from undetected CMHs [69, 70]. Current routine MRI sequences may not always be sensitive enough to detect these small hemorrhages, particularly soon after their occurrence when their effects on gait and behavior are most pronounced. This underscores the need for advances in imaging technologies and methodologies that can capture these subtle yet significant events. Understanding the nuanced dynamics of CMHs and their broader implications for brain function necessitates a deeper exploration of the stroke penumbra concept as it applies to these ministrokes, as well as a reconsideration of how we interpret transient neurological events and their potential underlying causes.

The observed strong correlation between stride time and stride length symmetry indices in our CMH model is particularly revealing. It highlights the intertwined nature of temporal and spatial aspects of gait, both of which are affected by the presence of CMHs. This interdependence suggests that changes in the coordination of movement are likely a direct consequence of CMH-induced brain damage, impacting both the planning and execution of locomotion. The alterations in gait symmetry indices provide a quantifiable reflection of these impacts, serving as a window into the subclinical stages of neurological impairment. The fluctuations in gait entropy offer additional insights into the complexity of gait dynamics in the presence of CMHs. The increase in gait entropy corresponding to periods of increased gait asymmetry signals a greater unpredictability in locomotion, potentially indicative of the brain's attempt to adapt to microhemorrhage-induced disruptions. Similarly, changes in the Regularity Index reveal alterations in inter-paw coordination, further emphasizing the widespread impact of CMHs on motor function.

Our findings have significant implications for the early detection of CMHs and potentially other neurological conditions. The sensitivity of gait analysis to the subclinical signs of neurological damage highlights its potential utility as a non-invasive screening tool. In particular, the ability to detect surges in gait asymmetry associated with CMHs points to the feasibility of using gait dynamics as an early indicator of microvascular brain damage in individuals at-risk, well before more overt clinical symptoms manifest.

The correlation between the number of CMHs and detected surges in gait asymmetry, although indicative of the sensitivity of our gait analysis, also suggests that the impact of CMHs on gait may depend on their specific locations and sizes. This finding hints at the complex interplay between the anatomical distribution of CMHs and their functional consequences, underscoring the need for further research to elucidate the mechanisms by which CMHs influence locomotion and to explore how these effects might vary based on the characteristics of the hemorrhages.

The incorporation of advanced gait metrics into the study of CMHs and CSVD in a preclinical setting significantly enhances our understanding of the nuanced impacts of CMHs on locomotion and behavior [25, 26, 36, 37, 71]. The cumulative incidence curves for neurological signs, as well as the detailed analyses of gait symmetry indices and entropy in our study, underscore the profound utility of these metrics in experimental research, particularly when examining between-group differences in susceptibility to CMH genesis. This approach is especially vital in studies aimed at evaluating the efficacy of treatments for the prevention of CMHs [25, 26]. One of the key strengths of utilizing advanced gait metrics as an outcome measure lies in their ability to provide a non-invasive, sensitive, and dynamic assessment of the neurological impact of CMHs over time. Given the challenges associated with longitudinal in vivo imaging for CMH detection—primarily due to limitations in resolution—gait analysis offers a practical and informative alternative. By tracking changes in gait dynamics, researchers can infer the presence and progression of CMHs and assess the neuroprotective efficacy of therapeutic interventions without the need for continuous imaging.

Moreover, the ability to detect subtle between-group differences in gait patterns allows for a more nuanced comparison of treatment effects, potentially revealing even minor improvements in neurological function that could signify a reduction in CMH formation or mitigation of their impact [25, 26]. This is particularly crucial in preclinical studies, where establishing the protective benefits of treatments against CMHs is a primary objective [25, 26]. The temporal resolution afforded by daily or frequent gait assessments enables researchers to identify critical periods of vulnerability or recovery, offering insights into the temporal efficacy of treatments. In summary, advanced gait metrics provide a powerful tool for experimental studies focused on CMHs, allowing for the sensitive detection of treatment effects and the exploration of susceptibility differences among groups. This approach not only enriches our understanding of the pathophysiology of CMHs but also opens new avenues for the development and evaluation of therapeutic strategies aimed at preventing these microvascular brain injuries. As the field progresses, the integration of these metrics with other neurological assessments will likely become an indispensable component of comprehensive research into CMHs and their prevention.

We would like to also highlight the potential of gait entropy as a pivotal metric for investigating CSVD across both preclinical and clinical settings [28, 72]. Integrating gait entropy with cognitive assessments offers a multifaceted approach to understanding the broader implications of CSVD on overall brain health [28]. This combination allows for comprehensive monitoring of both cognitive functions and motor abilities, which are often concurrently affected in CSVD. In clinical practice, leveraging gait entropy could enhance early detection strategies, enabling clinicians to identify neurological deterioration before significant cognitive decline becomes evident. This could lead to earlier interventions and more personalized treatment plans.

One notable limitation of our study is the inherent challenge in directly correlating specific gait alterations with the precise location and size of CMHs without extensive in vivo imaging capabilities. Future studies should aim to expand on these findings by incorporating more sophisticated imaging and analytical techniques to better understand the relationship between CMH characteristics (e.g., location, size) and specific gait alterations. While advanced gait metrics provide valuable insights into the neurological impact of CMHs, they offer an indirect measure of the underlying brain pathology. Consequently, interpreting changes in gait dynamics as specific indicators of CMH presence or progression requires cautious extrapolation, as these alterations could also be influenced by a variety of other factors not directly related to CMHs.

Furthermore, our reliance on a hypertensive mouse model to induce CMHs, while effective, may not fully encapsulate the complexity of CMH genesis in humans, where a wider array of risk factors and comorbidities play a role. This model-specific limitation highlights the broader challenge of translating findings from animal studies to human contexts. However, it is important to note that our recent human study suggests that CMHs are indeed associated with subclinical gait alterations [28], providing preliminary evidence that the gait metrics identified in our mouse model may have relevance in clinical populations. This finding underscores the potential utility of these gait metrics as biomarkers for CMHs in humans, albeit further research is necessary to validate their applicability and to understand the implications of various risk factors and comorbidities on gait dynamics in the context of CMHs. The translation of these findings from animal models to human studies represents a crucial step towards the development of non-invasive diagnostic and monitoring tools for CMHs and their associated neurological impacts. Additionally, the exclusion of female mice in our study limits the generalizability of our findings, as sex-specific responses to CMHs and their impact on gait dynamics were not explored, highlighting a critical area for future research.

 In conclusion, our study demonstrates the profound impact of hypertension-induced CMHs on gait dynamics in mice, offering valuable insights into the potential of gait analysis as a tool for early detection of neurological damage. These findings lay the groundwork for future research aimed at improving our understanding of CMHs and developing effective strategies for early diagnosis and intervention.
